# Rad51 Polymerization Reveals a New Chromatin Remodeling Mechanism

**DOI:** 10.1371/journal.pone.0003643

**Published:** 2008-11-04

**Authors:** Pauline Dupaigne, Christophe Lavelle, Anthony Justome, Sophie Lafosse, Gilles Mirambeau, Marc Lipinski, Olivier Piétrement, Eric Le Cam

**Affiliations:** 1 CNRS, Laboratoire de Microscopie Moléculaire et Cellulaire, UMR 8126 Interactions Moléculaires et Cancer, Institut de cancérologie Gustave Roussy, Villejuif, France; 2 Univ. Paris-Sud, Villejuif, France; 3 Division de Biochimie, UFR des Sciences de la Vie, Univ. Pierre et Marie Curie, Paris, France; Duke University, United States of America

## Abstract

Rad51 protein is a well known protagonist of homologous recombination in eukaryotic cells. Rad51 polymerization on single-stranded DNA and its role in presynaptic filament formation have been extensively documented. Rad51 polymerizes also on double-stranded DNA but the significance of this filament formation remains unclear. We explored the behavior of *Saccharomyces cerevisiae* Rad51 on dsDNA and the influence of nucleosomes on Rad51 polymerization mechanism to investigate its putative role in chromatin accessibility to recombination machinery. We combined biochemical approaches, transmission electron microscopy (TEM) and atomic force microscopy (AFM) for analysis of the effects of the Rad51 filament on chromatinized templates. Quantitative analyses clearly demonstrated the occurrence of chromatin remodeling during nucleoprotein filament formation. During Rad51 polymerization, recombinase proteins moved all the nucleosomal arrays in front of the progressing filament. This polymerization process had a powerful remodeling effect, as Rad51 destabilized the nucleosomes along considerable stretches of DNA. Similar behavior was observed with RecA. Thus, recombinase polymerization is a powerful mechanism of chromatin remodeling. These remarkable features open up new possibilities for understanding DNA recombination and reveal new types of ATP-dependent chromatin dynamics.

## Introduction

Rad51 is a key recombinase in the recombination process occurring during DNA repair, replication-fork rescue, meiotic chromosome segregation and telomere maintenance [1]. Early in homologous recombination, the nucleolytic processing of DNA double-stranded (ds) breaks or single-stranded (ss) gaps formed during replication produces a stretch of ss DNA that is targeted by recombinases and subsequently paired with a homologous duplex to form a DNA joint called a D-loop [2]. Like RecA in bacteria and RadA in Archaea, the eukaryote Rad51 recombinase promotes an ATP-mediated strand-exchange reaction by polymerizing on DNA and forming a helical filament. The formation of this presynaptic filament involves two steps: nucleation and extension. Rad51 is known to assemble into filamentous structures on ss DNA, thereby catalyzing homologous recombination. However, it has also been reported to polymerize on ds DNA [3]. The significance of this binding remains unclear, but Rad51 has very similar affinities for ss DNA and ds DNA [4]. Moreover chromatin is the physiological template for all DNA processing events in eukaryotes, raising questions about how homologous recombination can occur in the presence of nucleosomes. While Rad51 has been shown to potentiate the effect of Rad54 in D-loop formation [5]–[7], this eukaryotic recombinase alone is sufficient to mediate a homology search within nucleosomal templates [8].

We used molecular microscopy techniques [9]–[11] to explore the function of *Saccharomyces cerevisiae* Rad51 on different chromatinized DNA templates. We report here, for the first time, a spectacular Rad51-dependent chromatin remodeling mechanism, suggesting that Rad51—potentially in concert with Rad54—is a proficient chromatin remodeler in recombination and providing insight into its possible role in recombination. The bacterial recombinase RecA behaves in a similar manner, suggesting a new more general chromatin remodeling activity of recombinases, in which a nucleoprotein filament physically pushes the nucleosomes along the strand.

## Results

### Rad51 polymerization on linear nucleosomal templates induces nucleosome eviction

We found that *S. cerevisiae* Rad51 preferentially bound ss DNA over ds DNA, as previously reported for *S. pombe* Rad51 [12], on a hybrid DNA substrate with a single-stranded tail (Figs. S1a,b,c,f). However, a small increase in Rad51 concentration was sufficient for Rad51 polymerization and complete coverage of the ds DNA segment (Fig. S1d). Moreover, Rad51 underwent nucleation directly on ds DNA when ss DNA was protected by yRPA (Fig. S1e). Thus, Rad51 underwent nucleation and cooperative polymerization almost as efficiently on ds DNA as on ss DNA. These findings raise questions about the functional relevance of Rad51 polymerization on ds DNA.

**Figure 1:**
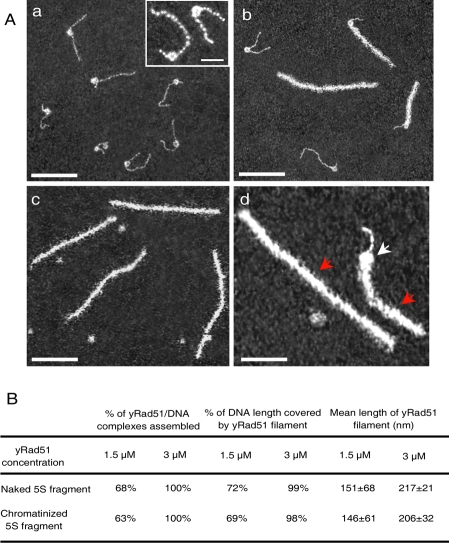
Rad51 polymerization on linear 5S nucleosomal templates. A. (a) Nucleosomal templates were reconstituted using a 420 bp DNA fragment with a 5S positioning sequence two thirds of the way from one end. Inset: enlarge image of single nucleosomes (scale bar: 25 nm). If Rad51 was added to a concentration of 1.5 µM (b) or 3 µM (c), Rad51 polymerization pushed the nucleosome, evicting it from the DNA fragment. (d) At a Rad51 concentration of 1.5 µM, the filament came into contact with nucleosomes (red and white arrows show Rad51 filament and nucleosome respectively). The scale bars represent 100 nm (a, b, c) and 50 nm (d). B. Comparison of Rad51 polymerization activity between naked and chromatinized 5S templates. The length of the naked 5S fragment was measured. The mean length obtained was 140±10 nm. Measurements were performed for 220 molecules.

As ds DNA is organized into chromatin *in vivo*, we investigated whether the polymerization of Rad51 on ds DNA was sufficiently powerful to induce chromatin remodeling. We used a 420 bp DNA fragment containing the *Lytechinus* 5S rDNA positioning sequence to generate mononucleosomal templates (Fig. 1Aa) [13]. At saturation ([Rad51]/[bp] ratio = 1/3), Rad51 polymerization had a powerful remodeling effect, which was accompanied by complete nucleosome eviction (Fig. 1Ac). To analyze the cooperativity of Rad51 on chromatinized substrate at lower ratios (see Figs 1Ab and 1Ad), we evaluate the percentage of Rad51 recovery, by measuring the length of the nucleofilament (Fig. 1B). No significant difference in the length of this filament was observed between naked and chromatinized DNA substrate, indicating that Rad51 nucleation and elongation were not altered by the presence of nucleosomes.

**Figure 2:**
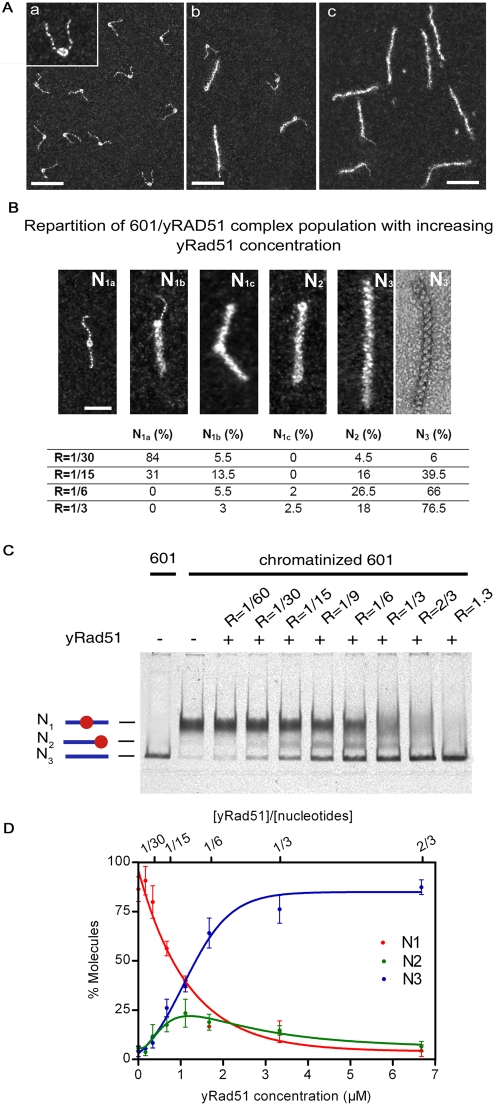
Rad51 polymerization on linear 601 nucleosomal templates. A. (a) Nucleosomal templates were reconstituted on a 601 positioning sequence located at the center of a 347 bp DNA fragment. (b–c) Despite the high affinity of the 601 sequence, Rad51 polymerization shifts or ejects nucleosomes. The scale bars represent 50 nm for all pictures (a,b,c). Inset: enlarge image of single nucleosome (zoom 2×). B. Classification of 601/Rad51 complexes (N_1a_, N_1b_, N_1c_, N_2_, N_3_) and distribution of the population with increasing [Rad51]/[bp] ratio. For the N_3_ complex, positive and negative staining pictures confirm nucleosome eviction. For all images, the scale bar represents 50 nm. C. Rad51 remodeling shift assay, showing the remodeling occurs from low Rad51 concentrations. D. Graph showing the efficiency of Rad51 remodeling activity as a function of concentration. Error bars are standard deviation of the mean.

We assessed the strength of this remodeling activity, using the stronger nucleosome positioning sequence, 601 (Fig 2Aa). The more homogeneous distribution of nucleosomes on the 601 fragment confirmed its higher affinity (the 601 positioning sequence binds the histone octamers 150 times more strongly than the 5S rDNA gene [14]). Again, at a [Rad51]/[bp] ratio of 1/3, Rad51 polymerization led to nucleosome removal (Fig. 2Ac), demonstrating the strength of this polymerization-induced remodeling mechanism. For lower [Rad51]/[bp] ratios, three intermediate states were observed, in which the Rad51 filament came into direct contact with nucleosomes (Fig. 2Ab): (1) filament blocked by the nucleosome (N_1b_), (2) nucleosome trapped between filaments originating from two nucleation points (N_1c_), and (3) nucleosome pushed to the end by Rad51 polymerization (N_2_). Figure 2B provides a classification with a quantitative representation of the distribution of the five classes of molecules (free 601 mononucleosomes (N_1a_), the three intermediates and molecules fully covered with Rad51 (N_3_)) with increasing [Rad51]/[bp] ratio. We considered N_2_ and N_3_ to be substrates that had undergone chromatin remodeling. At a low [Rad51]/[bp] ratio (1/30), more than 10% of the chromatinized 601 was remodeled, pointing out the high level of cooperativity of Rad51 polymerization, even in the presence of nucleosome. Regardless of the stoichiometric conditions, the small size of the N_1c_ population—characterized by two points of nucleation—provided evidence for the high speed of Rad51 polymerization. Finally, beyond the saturation ratio, most molecules were remodeled (about 60% for R = 1/15 and more than 90% for R = 1/6). At saturation, we analyzed the structure of Rad51 filaments using negative staining TEM experiments (see Fig. 2B) in order to confirm the eviction of nucleosome. We then measured an average length of 183±25 nm and a pitch value of 9.5±0.5 nm in agreement with reported values [15], excluding the presence of nucleosome inside the filament. In parallel, we developed a PAGE remodeling assay based on destabilization of the Rad51 filament by EDTA just after Rad51 polymerization on the 601 mononucleosome substrate. The results obtained in the PAGE Rad51 remodeling assay were consistent with all the quantitative results obtained in TEM experiments (Figs. 2C and 2D).

From these experiments, it remained unclear how the nucleosomes are ejected from the DNA, as this new “polymerization-induced” remodeling mechanism might involve Rad51 either disrupting the nucleosomes or pushing them beyond the template.

### Rad51 polymerization on circular nucleosomal templates induces nucleosome sliding

**Figure 3:**
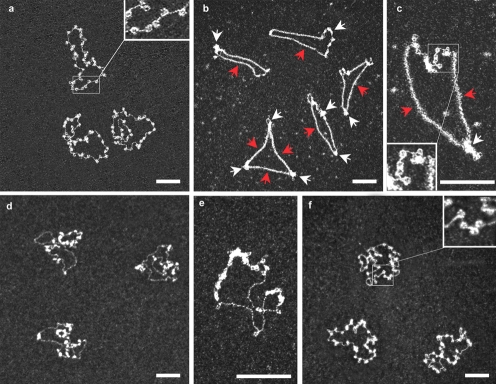
Rad51 polymerization on circular nucleosomal template. Rad51 unwinds DNA and destabilizes entire nucleosome arrays in a partially reversible fashion. (a) Chromatin was reconstituted on the ΦX174 supercoiled plasmid, giving an array of 30 to 35 nucleosomes. Inset: enlarge image of nucleosomes (zoom 2.5×); (b,c) when Rad51 is added, 2 to 3 filaments are generally formed (from 2 to 3 nucleation sites, probably starting in the linker DNA between nucleosomes), stretching over several hundred bp on straight nucleosome-free DNA and pushing nucleosomes into 2 to 3 dense arrays. . (red and white arrows show Rad51 filament and nucleosome clusters, respectively) Inset in (c): enlarge image of nucleosomes compacted by Rad51 filament (zoom 1.5×) ; (d,e) subsequent addition of EDTA to a high concentration destabilizes Rad51 filaments, allowing supercoiled nucleosome arrays to relax; (f) further treatment at 40°C for 20 minutes leads to spontaneous nucleosome sliding, making it possible to check for nucleosome loss during the partially reversible remodeling process. Inset: enlarge image of nucleosomes after thermal redistribution (zoom 2.5×). The scale bars represent 100 nm for all pictures.

We investigated whether the Rad51 filament pushed or disrupted nucleosomes, by carrying out remodeling assays on circular chromatinized templates. Nucleosome arrays were assembled on the ΦX174 RFI (Replication Form I) 5386 bp supercoiled plasmid. We counted a mean of 31±4 nucleosomes per reconstituted template (Fig. 3a). The addition of Rad51 (3 µM) to this chromatinized template resulted in the formation of polymers from two or three nucleation sites, separated by constrained clusters of nucleosomes (Figs. 3b and 3c). We investigated the number and integrity of the nucleosomes remaining on the plasmids, by setting up a “reverse remodeling” assay: (1) we first dissociated Rad51 filaments completely from the templates by incubation with a high concentration of EDTA, leaving the nucleosomes compacted in discrete arrays (Figs. 3d and 3e). This indicated that most nucleosomes had indeed being driven to slide on the DNA during the initial remodeling step. (2) We then induced nucleosome sliding by incubation for 20 minutes at 40°C, leading to the redistribution of nucleosomes along the templates (Fig. 3f). Our analysis of ∼200 molecules indicated that 23±3 of the 31±4 nucleosomes initially present on the plasmids remained bound after remodeling step, with the remaining nucleosomes ejected. This finding confirmed the strong remodeling effect of Rad51 polymerization and its unique capacity to shift whole nucleosome arrays along the template. This is the first direct observation of multi-nucleosome remodeling along several hundreds of nanometers triggered by the progression of a nucleoprotein filament.

### AFM analysis demonstrates that Rad51 filaments formed on constrained nucleosomal templates have the same pitch as those formed on unconstrained naked DNA

**Figure 4:**
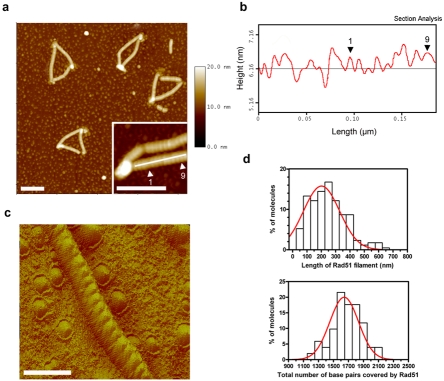
Rad51 filament structure on circular nucleosomal template. Rad51 polymerization on nucleosome arrays was imaged by AFM to assess the structure of the nucleoprotein filament in a “chromatin-constrained” context. (a,b) The images obtained were similar to those for EM observation (compare with Fig. 3b). However, AFM allows the direct measurement of filament parameters (see inset and corresponding profile in b),confirming the ∼9.4 nm±0.6 (∼19 bp, as deduced from total covering) helical pitch previously reported for linear ds DNA-Rad51 filaments. (c) The 3D view even clearly shows the right-handed helicoid filament formed by Rad51 polymerization on ds DNA. (d) The total coverage of circular substrates and individual Rad51 filament lengths were obtained by image analysis for more than 200 molecules. The scale bars represent 100 nm (a) and 50 nm (c).

The Rad51 filaments formed on unconstrained ds templates progress freely, unwinding the double helix (∼19 vs. 10.5 bp per helical turn) and stretching the DNA by a factor of 1.5 [15]. However, Rad51 polymerization is blocked by contact with closely packed nucleosome arrays on chromatinized templates (Fig. 3d and 4a) or by topological torque on naked closed circular plasmids (Fig. S2). We used high-resolution AFM imaging to measure the helical pitch of the filament in these conditions and compared this pitch to that for a canonical filament formed on relaxed DNA. In all cases, the DNA was found to be stretched and unwound to the same extent, with a right-handed helical pitch observed and analyzed on 3D images (Fig. 4c). The Rad51 filament structure is thus robust and invariant, whether formed in the presence or absence of constraints.

The distribution of Rad51 filament lengths also demonstrated that the remodeling process was efficient (Fig. 4d). Nucleosome arrays were pushed up to 600 nm—corresponding to more than 1200 bp—as deduced from the helical pitch value. We investigated whether the same remodeling mechanism occurred on different chromatin templates, by also studying the effect of Rad51 on long native chromatin fibers. Again, Rad51 polymerization provoked extensive remodeling activity affecting several adjacent nucleosomes (Fig. S3). Values for total coverage of the template and pitch were used to estimate the topological constraints generated by the polymerization process (Fig. 4). By untwisting several hundreds of bp of DNA, the Rad51 filament induces strong positive supercoiling of the rest of the template. On closed circular naked plasmids, this constraint is concentrated in very densely supercoiled plectonemic regions (Fig. S2). On circular nucleosome arrays (Fig. 3 and Fig. 4), a major change in chromatin organization and/or nucleosome conformation is required to absorb the topological constraint confined within such clusters. Indeed, a basic estimation of the topological state of the DNA within these clusters gives a surprisingly high ΔLk, at >+1 per nucleosome, whereas “canonical” nucleosomes have a ΔLk∼−1 [16]. Compact chromatin clusters probably adopt particularly highly supercoiled conformations (see the striking height of these clusters measured by AFM). Alternatively, nucleosomes may undergo structural changes related to the chirally reversed nucleosome (“reversome”) recently proposed based on single-molecule studies [17].

### RecA recombinase also displays remodeling activity, although this activity is weaker than that of Rad51

The bacterial RecA recombinase polymerizes on both ss DNA and ds DNA in the presence of adenosine 5′-triphosphate (ATP), forming helical nucleoprotein filaments. RecA polymers are, however, known to be less stable on ds DNA than on ss DNA. As RecA dissociation depends exclusively on ATP hydrolysis, we therefore considered the polymerization process and used ATPγS.

Chromatin remodeling experiments were carried out on 5S, 601 and phiX174 RFI DNA substrates. At a ratio [RecA]/[bp] of 1/3, RecA polymerization on ds DNA left to the shifting of nucleosomes, leading to their eviction from the 5S substrate, as observed for Rad51 (Fig. S4Aa), thereby demonstrating remodeling activity. However, RecA filaments were often blocked by the positioning of nucleosomes on the 601 sequence (Fig. S4Ab), suggesting that RecA polymerization was less efficient than Rad51 in chromatin remodeling. This finding was confirmed by studies on the chromatinized phiX174 RFI (Fig. S4Ac), which displayed shorter RecA filaments associated with naked DNA and nucleosome clusters.

As for Rad51, we present a quantitative distribution of the same five classes of molecules with increasing [RecA]/[bp] ratio (Fig. S4B). This analysis confirmed the lower efficiency of RecA polymerization than of Rad51 polymerization on ds DNA, as only 45% of the molecules remained uncovered at a ratio of 1/3. The N_1b_ and N_1c_ populations were therefore larger than those for Rad51, possibly due to the lower efficiency of RecA remodeling and the binding properties of RecA on ds DNA. Indeed, RecA polymerization was sensitive to nucleosome positioning sequence affinity, whereas Rad51 remodeling activity was similar for all affinities of the nucleosome positioning sequence (Figs. 1 and 2). The high proportion of nucleosomes in the state may be accounted for by several nucleation points for RecA [18] and by nucleosome shift occurring more slowly than nucleation and filament growth.

## Discussion

### Rad51 polymerization on ds DNA

Recombinases from the three kingdoms of life (RecA, RadA and Rad51 from prokaryotes, archaea and eukaryotes, respectively) assemble on both ss and ds DNA, forming very similar filaments, each requiring a bound nucleotide cofactor [19]. Rad51 polymerizes faster on ss DNA, but the filaments it forms on ds DNA are more stable and depolymerize much more slower than those formed on ss DNA [20]. The non cooperative ATPase activity of Rad51 [21] makes Rad51 filaments less dynamic than RecA filaments, potentially resulting in dead-end complexes on undamaged DNA or after strand exchange [22]. This highlights the essential role of the partners of Rad51 in facilitating depolymerization and regulating recombination [11], [23]. Filament formation requires ATP binding, but ATP hydrolysis is required only for filament destabilization and turnover [19], [24]. This may explain why ATP hydrolysis is dispensable for the recombination reactions catalyzed by Rad51 *in vitro* (assessed mostly by joint molecule formation) but indispensable for recombination *in vivo* [25]. Indeed, efficient Rad51 turnover from ds DNA has recently been shown to require the ATPase activities of both Rad51 and Rad54 [22].

### A new remodeling mechanism

Many ATP-dependent enzymes alter chromatin structure during gene transcription and other processes involving DNA [26]. Most of the remodeling molecules identified to data seem to be translocases, their ATP-dependent translocation activity resulting in changes in nucleosome positioning and structure [27]. A number of mechanisms have been proposed to account for nucleosome remodeling events: bulge diffusion, twist diffusion and partial nucleosome unwrapping, generally involving a single remodeling complex on a single nucleosome at a time [27]. The chromatin remodeling activity described here is different in that it depends on a polymerization process that physically pushes whole nucleosome arrays along the DNA, destabilizing them. Other enzymes tracking and unwinding DNA may also induce local disturbances in eukaryotic chromatin structure, as reported for various polymerases [28] and some prokaryotic helicases [29], [30]. DNA twisting and/or unwinding activity alone is not sufficient to induce nucleosome sliding, as shown by single-molecule experiments on chromatin fibers [17] or ethidium bromide intercalation assays on mononucleosomes [31]. In both cases, the high stability of nucleosomes under supercoiling constraints appears to be due to its ability to either positively or negatively cross the entry/exit DNAs [31], [32]. Thus, induced topological stress may lead nucleosomes to a transient metastable state, which may facilitate remodelers activity. Polymerization is a powerful mechanism of protein progression along DNA and may facilitate nucleosome remodeling events. The Rad51 recombinase is the first protein shown to be involved in chromatin remodeling through an ATP-fuelled dynamic polymerization process. This collective remodeling capability is unusually strong among known remodeling factors.

The Rad51 concentration used in our experiments was that used in strand exchange assays, consistent with the widely accepted *in vivo* function of presynaptic filament formation and the results of homology searches. Our results thus demonstrate that physiological amounts of Rad51 can displace nucleosomes through Rad51 polymerization and strong cooperative binding. The polymerization strength of a few Rad51 proteins on a short ds DNA segment is great enough to induce a powerful remodeling effect on a few nucleosomes (Figs. 2C & 2D). Given this new role of Rad51, this protein may be considered to be a molecular motor, as suggested for RecA and its multiple ATP-dependent activities in bacterial recombination [33]. However, as stated above, Rad51 polymerization is induced exclusively by ATP [24], making a “one shot” remodeling action without ATP hydrolysis possible. Rad51 destabilization on ds DNA and turnover then require the ATPase activities of both Rad51 and Rad54 [22]. The eukaryotic equivalent of RecA may therefore be seen as the equivalent of a Rad51/Rad54 combination rather than as Rad51 alone. Rad54 enables Rad51 to form a D-loop with a chromatinized DNA template—even more efficiently than with naked DNA template in some cases [6] indicating that these two eukaryotic factors may have evolved together to deal with the various steps of recombination in the context of chromatin, their natural substrate *in vivo*.

### Recombination in the context of chromatin

Rad54 is considered as the main partner of Rad51 in the regulation of homologous recombination in the context of chromatin. Rad54 belongs to the Swi2/Snf2 family and induces a limited nucleosome sliding [5], [7], [34]. It remains unclear whether such remodeling activity is part of the normal attributes of the pleiotropic Rad54 molecule [35]. It has been reported that efficient remodeling occurs only in the presence of both Rad51 and Rad54, suggesting that Rad51 enhances the remodeling activity of Rad54 [6], [36].

Homologous recombination requires an initial search of sequence homology and a subsequent strand invasion. A recent study [8] reports that Rad51 presynaptic filament is sufficient to mediate efficiently a homology search on chromatinized template leading to the formation of paranemic joints, which is consistent with the strong remodelling activity of Rad51 we described here. yRad54 converts these initial joints into stable plectonemic joints called D-loop.

The major chromatin remodeling activity over several hundred bp induced by Rad51 polymerization shown here may promote D-loop extension. Rad54 cooperates with Rad51 throughout the entire process, consistent with the known requirement for this molecule for DNA synthesis after synapsis *in vivo* [37] and with its association with the terminus of the Rad51-ds DNA filament, [38] possibly resulting in the destabilization of Rad51 at the end of the remodeling process [39], [40]. Interestingly, whereas Rad51/Rad54 together gave efficient D-loop formation on chromatin template *in vitro*, this activity was abolished if Rad51 was replaced by RecA [6]. This may be due to a lack of functional cooperation between RecA and Rad54 activities in recombination and confirms that Rad54-induced remodeling alone is not sufficient for correct recombination in chromatin. Indeed, Rad54 remodeling activity, which is thought to involve an ISWI-like mechanism [41], seems to be far less striking than the Rad51 “polymerization-induced” remodeling mechanism reported here. Our results thus reveal a new feature in the synergy operating between Rad54 and Rad51 in chromatin remodeling.

## Materials and Methods

### DNA templates

A 420 bp 5S DNA fragment containing the *L. variegatus* 5S rDNA sequence [42] was obtained by PCR amplification from a pUC(357) plasmid. The fluorescent 349 bp 601 fragment was obtained by PCR amplification from a pGEM plasmid including the 601 nucleosome positioning sequence, kindly provided by Jonathan Widom, using a Cy5-labeled primer (all primers are listed in table S1). All DNA products were finally purified on a MiniQ anion exchange column with SMART system chromatography (GE Healthcare).

### Chromatin reconstitution

Chromatin was assembled on the various DNA templates by exchange in high salt conditions with purified core particles (CP) from calf thymus (see [43] and references therein). Briefly, chromatin was extracted in low ionic strength buffer after the digestion of nuclei with micrococcal nuclease;, core particles were obtained by further digestion with micrococcal nuclease and purified by chromatography on a Sephacryl S300 HR column (Pharmacia Biotech). The mononucleosome fraction was concentrated by ultrafiltration in a pressurized cell (model 8003, Amicon) and stored at 0°C. Sodium dodecyl sulfate-polyacrylamide gel electrophoresis was carried out in a 15% acrylamide gel, to analyze histone composition. For reconstitution, equimolar amounts of core particles and DNA fragments were mixed in 2 M NaCl and subjected to successive dilutions (to 1 M, 0.8 M and 0.6 M NaCl). They were then dialyzed against 10 mM TriS-HCl pH 7.5, 300 mM NaCl, and then against 10 mM TriS-HCl pH 7.5, 100 mM NaCl. Reconstituted chromatin was purified from the remaining free core particles and free core DNA fragments on a Superose 6B column with a SMART system (GE Healthcare). Mononucleosomes reconstituted on the 601 sequence were separated from the 146 bp DNA fragment on a Superdex200 PC3.2/30 column (GE Healthcare).

### Rad51 binding assays

*Saccharomyces cerevisiae* Rad51 protein was overproduced in *E. coli* BL21 (DE3) pLysS cells carrying pEZ5139 (kindly provided by Dr. Steve Kowalczykowski) and was purified as previously described. For Rad51 binding assays, 10 µM of naked DNA or chromatinized DNA substrate was incubated with various amount of Rad51 (0.5 to 10 µM) for 15 min at 37°C, in a buffer containing 10 mM Tris-HCl (pH 7.5), 50 mM NaCl, 5 mM MgCl_2_ and 1.5 mM ATP. In the Rad51 destabilization assay, we added 50 mM EDTA to the mixture just after the polymerization step described above, and unbound Rad51 protein was eliminated by gel filtration on a SMART system. For thermal redistribution, the EDTA reaction products were incubated for 20 minutes at 40°C.

### Rad51 remodeling assay

We incubated Rad51 with mononucleosomes reconstituted on Cy5-labeled 601 fragment in the conditions described above and then destabilized by incubation with 50 mM EDTA. Samples were loaded onto a 4% polyacrylamide gel and run in 0.2×TBE for 60 minutes at 75 V. Gels were scanned using a Fuji FLA-3000 PhosphorScreen and the bands were quantified using ImageQuant Software.

### TEM and AFM experiments

#### TEM positive staining

5 µl of reaction product was diluted to 1 to 5 nM in 10 mM Tris-HCl pH 7.5, 5 mM MgCl_2_, 50 mM NaCl and deposited on a 600-mesh copper grid covered with a thin carbon film, activated by glow-discharge in the presence of pentylamine [11]. Grids were washed with aqueous 2% (w/v) uranyl acetate, dried and observed in the annular dark-field mode, using a Zeiss 902 transmission electron microscope. Images were captured at a magnification of 85,000× or 140,000× with a MegaviewIII CCD camera and analyzed with iTEM software (Olympus Soft Imaging Solution).

#### TEM negative staining

Samples were prepared as describes above, and were applied to glow-discharged carbon-coated copper grids and negatively stained with 2% uranyl acetate. Grids were observed, at a magnification of 140,000×, in bright field mode using a Zeiss 902 transmission electron microscope.

#### AFM

Rad51-ds DNA nucleoprotein filaments, in presence or absence of nucleosomes, were diluted 1/30 in 10 mM Tris pH 7.5, 50 mM NaCl, 5 mM MgCl_2_ and 100 µM spermidine [9], [44]. A 5 µl droplet of the resulting solution was deposited onto the surface of freshly cleaved muscovite for 1 minute. The surface was rinsed with buffer, and the sample dried. Imaging was carried out in Tapping Mode™, with a Multimode™ system (Veeco) operating with a Nanoscope IIIa™ controller (Veeco). We used silicon AC160TS cantilevers (Olympus) with resonance frequencies of about 300 kHz. All images were collected at a scan frequency of 1.5 Hz and a resolution of 512×512 pixels. Images were analyzed with Nanoscope software. A second-order polynomial function was used to remove the background. No gluteraldehyde fixation was used for either AFM or TEM. All data were collected from series of at least 200 molecules.

## Supporting Information

Figure S1 Rad51 polymerization on naked linear DNA templates. Rad51 polymerizes on ss and ds DNA in a sequential fashion, first entering ss and then covering ds. Covering of the ss-ds DNA construction with 0.5 µM (a), 1 µM (b), 2 µM (c), 3 µM (d) and same conditions+1 µM RPA (e). The scale bars represent 100 nm for all pictures. (f) Rad51 covering of the ds/ss hybrid substrate (609 bp of ds DNA+831 nt of ss DNA). Note that mainly 100% covering is obtained on ss as well as ds DNA in 3 µM Rad51.
(12.89 MB TIF)

Figure S2 Rad51 polymerization on a naked plasmid. When Rad51 polymerizes on a closed circular naked plasmid (see naked control in a), high positive constraint concentrates in very densely positively supercoiled plectonemic regions (b). When relaxation is allowed during the reaction (c: see relaxed naked plasmid for comparison), filaments cover the whole plasmid (d). As in Fig. 4, AFM allows us to directly measure filament parameters (e: see insert in the picture and profile below). Total covering of the circular substrates and individual Rad51 filament lengths were obtained from image analysis of more than 200 molecules series from TEM and AFM pictures (f). The scale bars represent 200 nm (a–d), 250 nm (e) and 50 nm (inset).
(10.07 MB TIF)

Figure S3 Rad51 polymerization on long native linear nucleosomal templates. When added on long native chromatin fibers (∼60 nucleosomes distributed on 11000 bp) (a), Rad51 polymerization induced chromatin remodeling of the same extent than the remodeling events observed on circular templates (b), confirming the robustness of this mechanism. Red and white arrows show Rad51 filament and nucleosome cluster, respectively. The scale bars represent 100 nm for all pictures.
(1.52 MB TIF)

Figure S4 RecA polymerization also displays remodeling activity. A. Nucleosomal templates were reconstituted on a 601 positioning sequence located at the center of a 347 bp DNA fragment (a). Despite the high affinity of the 601 sequence, Rad51 polymerization shifts or ejects nucleosomes. The scale bars represent 50 nm for all pictures. B. Distribution of the 601/Rad51 complex population as a function of increasing [RecA]/[bp] ratio. All pictures were acquired at the same magnification and the scale bare represents 50 nm.
(13.05 MB TIF)

Table S1 Sequence of primers used for PCR.
(0.03 MB DOC)

## References

[1] Sung P, Klein H (2006). Mechanism of homologous recombination: mediators and helicases take on regulatory functions.. Nat Rev Mol Cell Biol.

[2] Sung P (1994). Catalysis of ATP-dependent homologous DNA pairing and strand exchange by yeast RAD51 protein.. Science.

[3] Zaitseva EM, Zaitsev EN, Kowalczykowski SC (1999). The DNA binding properties of Saccharomyces cerevisiae Rad51 protein.. J Biol Chem.

[4] Dupaigne P, Le Breton C, Fabre F, Gangloff S, Le Cam E (2008). The Srs2 helicase activity is stimulated by Rad51 filaments on dsDNA: implications for crossover incidence during mitotic recombination.. Molecular Cell.

[5] Jaskelioff M, Van Komen S, Krebs JE, Sung P, Peterson CL (2003). Rad54p is a chromatin remodeling enzyme required for heteroduplex DNA joint formation with chromatin.. J Biol Chem.

[6] Alexiadis V, Kadonaga JT (2002). Strand pairing by Rad54 and Rad51 is enhanced by chromatin.. Genes Dev.

[7] Alexeev A, Mazin A, Kowalczykowski SC (2003). Rad54 protein possesses chromatin-remodeling activity stimulated by the Rad51-ssDNA nucleoprotein filament.. Nat Struct Biol.

[8] Sinha M, Peterson CL (2008). A Rad51 presynaptic filament is sufficient to capture nucleosomal homology during recombinational repair of a DNA double-strand break.. Molecular Cell.

[9] Hamon L, Pastre D, Dupaigne P, Le Breton C, Le Cam E (2007). High-resolution AFM imaging of single-stranded DNA-binding (SSB) protein–DNA complexes.. Nucleic Acids Res.

[10] Mortier-Barriere I, Velten M, Dupaigne P, Mirouze N, Pietrement O (2007). A key presynaptic role in transformation for a widespread bacterial protein: DprA conveys incoming ssDNA to RecA.. Cell.

[11] Veaute X, Jeusset J, Soustelle C, Kowalczykowski SC, Le Cam E (2003). The Srs2 helicase prevents recombination by disrupting Rad51 nucleoprotein filaments.. Nature.

[12] Sauvageau S, Stasiak AZ, Banville I, Ploquin M, Stasiak A (2005). Fission Yeast Rad51 and Dmc1, two efficient DNA recombinases forming helical nucleoprotein filaments.. Mol Cell Biol.

[13] Simpson RT, Stafford DW (1983). Structural features of a phased nucleosome core particle.. Proc Natl Acad Sci U S A.

[14] Thastrom A, Lowary PT, Widlund HR, Cao H, Kubista M (1999). Sequence motifs and free energies of selected natural and non-natural nucleosome positioning DNA sequences.. J Mol Biol.

[15] Yu X, Jacobs SA, West SC, Ogawa T, Egelman EH (2001). Domain structure and dynamics in the helical filaments formed by RecA and Rad51 on DNA.. Proc Natl Acad Sci U S A.

[16] Prunell A (1998). A topological approach to nucleosome structure and dynamics: the linking number paradox and other issues.. Biophys J.

[17] Bancaud A, Wagner G, Conde ESN, Lavelle C, Wong H (2007). Nucleosome chiral transition under positive torsional stress in single chromatin fibers.. Mol Cell.

[18] Galletto R, Amitani I, Baskin RJ, Kowalczykowski SC (2006). Direct observation of individual RecA filaments assembling on single DNA molecules.. Nature.

[19] Ristic D, Modesti M, van der Heijden T, van Noort J, Dekker C (2005). Human Rad51 filaments on double- and single-stranded DNA: correlating regular and irregular forms with recombination function.. Nucleic Acids Res.

[20] van der Heijden T, Seidel R, Modesti M, Kanaar R, Wyman C (2007). Real-time assembly and disassembly of human RAD51 filaments on individual DNA molecules.. Nucleic Acids Res.

[21] Tombline G, Fishel R (2002). Biochemical characterization of the human RAD51 protein. I. ATP hydrolysis.. J Biol Chem.

[22] Li X, Zhang XP, Solinger JA, Kiianitsa K, Yu X (2007). Rad51 and Rad54 ATPase activities are both required to modulate Rad51-dsDNA filament dynamics.. Nucleic Acids Res.

[23] Symington LS, Heyer WD (2006). Some disassembly required: role of DNA translocases in the disruption of recombination intermediates and dead-end complexes.. Genes Dev.

[24] Chi P, Van Komen S, Sehorn MG, Sigurdsson S, Sung P (2006). Roles of ATP binding and ATP hydrolysis in human Rad51 recombinase function.. DNA Repair (Amst).

[25] Stark JM, Hu P, Pierce AJ, Moynahan ME, Ellis N (2002). ATP hydrolysis by mammalian RAD51 has a key role during homology-directed DNA repair.. J Biol Chem.

[26] Saha A, Wittmeyer J, Cairns BR (2006). Chromatin remodelling: the industrial revolution of DNA around histones.. Nat Rev Mol Cell Biol.

[27] Flaus A, Owen-Hughes T (2004). Mechanisms for ATP-dependent chromatin remodelling: farewell to the tuna-can octamer?. Curr Opin Genet Dev.

[28] Lavelle C (2007). Transcription elongation through a chromatin template.. Biochimie.

[29] Eggleston AK, O'Neill TE, Bradbury EM, Kowalczykowski SC (1995). Unwinding of nucleosomal DNA by a DNA helicase.. J Biol Chem.

[30] Ramsperger U, Stahl H (1995). Unwinding of chromatin by the SV40 large T antigen DNA helicase.. EMBO J.

[31] Sivolob A, De Lucia F, Revet B, Prunell A (1999). Nucleosome dynamics. II. High flexibility of nucleosome entering and exiting DNAs to positive crossing. An ethidium bromide fluorescence study of mononucleosomes on DNA minicircles.. J Mol Biol.

[32] Bancaud A, Conde e Silva N, Barbi M, Wagner G, Allemand JF (2006). Structural plasticity of single chromatin fibers revealed by torsional manipulation.. Nat Struct Mol Biol.

[33] Cox MM (2007). Motoring along with the bacterial RecA protein.. Nat Rev Mol Cell Biol.

[34] Wolner B, Peterson CL (2005). ATP-dependent and ATP-independent roles for the Rad54 chromatin remodeling enzyme during recombinational repair of a DNA double strand break.. J Biol Chem.

[35] Heyer WD, Li X, Rolfsmeier M, Zhang XP (2006). Rad54: the Swiss Army knife of homologous recombination?. Nucleic Acids Res.

[36] Kwon Y, Chi P, Roh DH, Klein H, Sung P (2007). Synergistic action of the Saccharomyces cerevisiae homologous recombination factors Rad54 and Rad51 in chromatin remodeling.. DNA Repair (Amst).

[37] Sugawara N, Wang X, Haber JE (2003). In vivo roles of Rad52, Rad54, and Rad55 proteins in Rad51-mediated recombination.. Mol Cell.

[38] Kiianitsa K, Solinger JA, Heyer WD (2006). Terminal association of Rad54 protein with the Rad51-dsDNA filament.. Proc Natl Acad Sci U S A.

[39] Bugreev DV, Hanaoka F, Mazin AV (2007). Rad54 dissociates homologous recombination intermediates by branch migration.. Nat Struct Mol Biol.

[40] Solinger JA, Kiianitsa K, Heyer WD (2002). Rad54, a Swi2/Snf2-like recombinational repair protein, disassembles Rad51:dsDNA filaments.. Mol Cell.

[41] Zhang Z, Fan HY, Goldman JA, Kingston RE (2007). Homology-driven chromatin remodeling by human RAD54.. Nat Struct Mol Biol.

[42] Sivolob A, Lavelle C, Prunell A (2003). Sequence-dependent nucleosome structural and dynamic polymorphism. Potential involvement of histone H2B N-terminal tail proximal domain.. J Mol Biol.

[43] Hayes JJ, Lee KM (1997). In vitro reconstitution and analysis of mononucleosomes containing defined DNAs and proteins.. Methods.

[44] Pastré D, Hamon L, Landousy F, Sorel I, David MO (2006). Anionic polyelectrolyte adsorption on mica mediated by multivalent cations: a solution to DNA imaging by atomic force microscopy under high ionic strengths.. Langmuir.

