# Supplementary figures and images for: Rad51 Polymerization Reveals a New Chromatin Remodeling Mechanism
Source: PLoS One. 2008 Nov 4;3(11):e3643. doi: 10.1371/journal.pone.0003643 (PMC2574414; doi:10.1371/journal.pone.0003643)

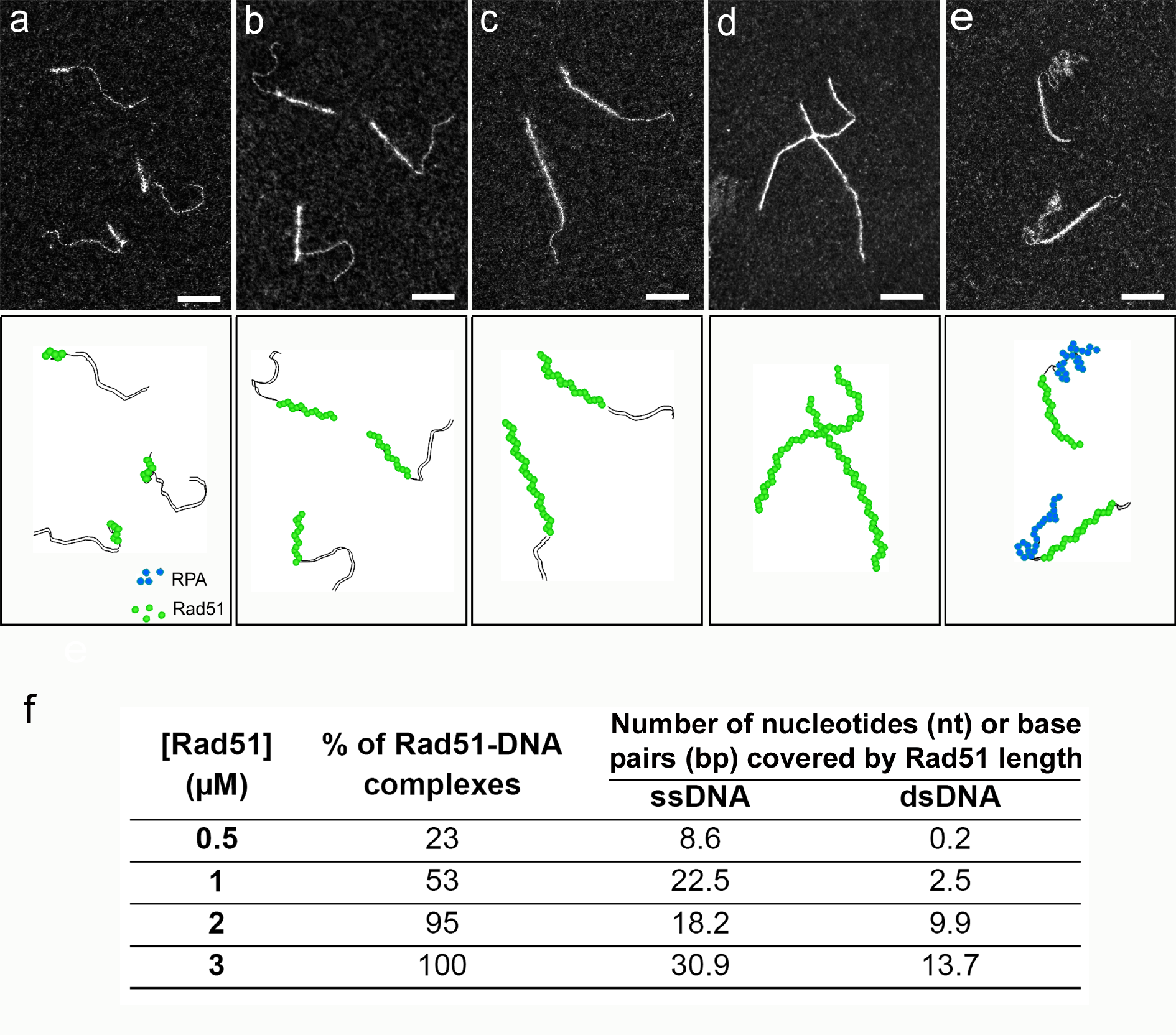

Supplement: Figure S1 — Rad51 polymerization on naked linear DNA templates. Rad51 polymerizes on ss and ds DNA in a sequential fashion, first entering ss and then covering ds. Covering of the ss-ds DNA construction with 0.5 µM (a), 1 µM (b), 2 µM (c), 3 µM (d) and same conditions+1 µM RPA (e). The scale bars represent 100 nm for all pictures. (f) Rad51 covering of the ds/ss hybrid substrate (609 bp of ds DNA+831 nt of ss DNA). Note that mainly 100% covering is obtained on ss as well as ds DNA in 3 µM Rad51. (12.89 MB TIF) [file pone.0003643.s001.tif]

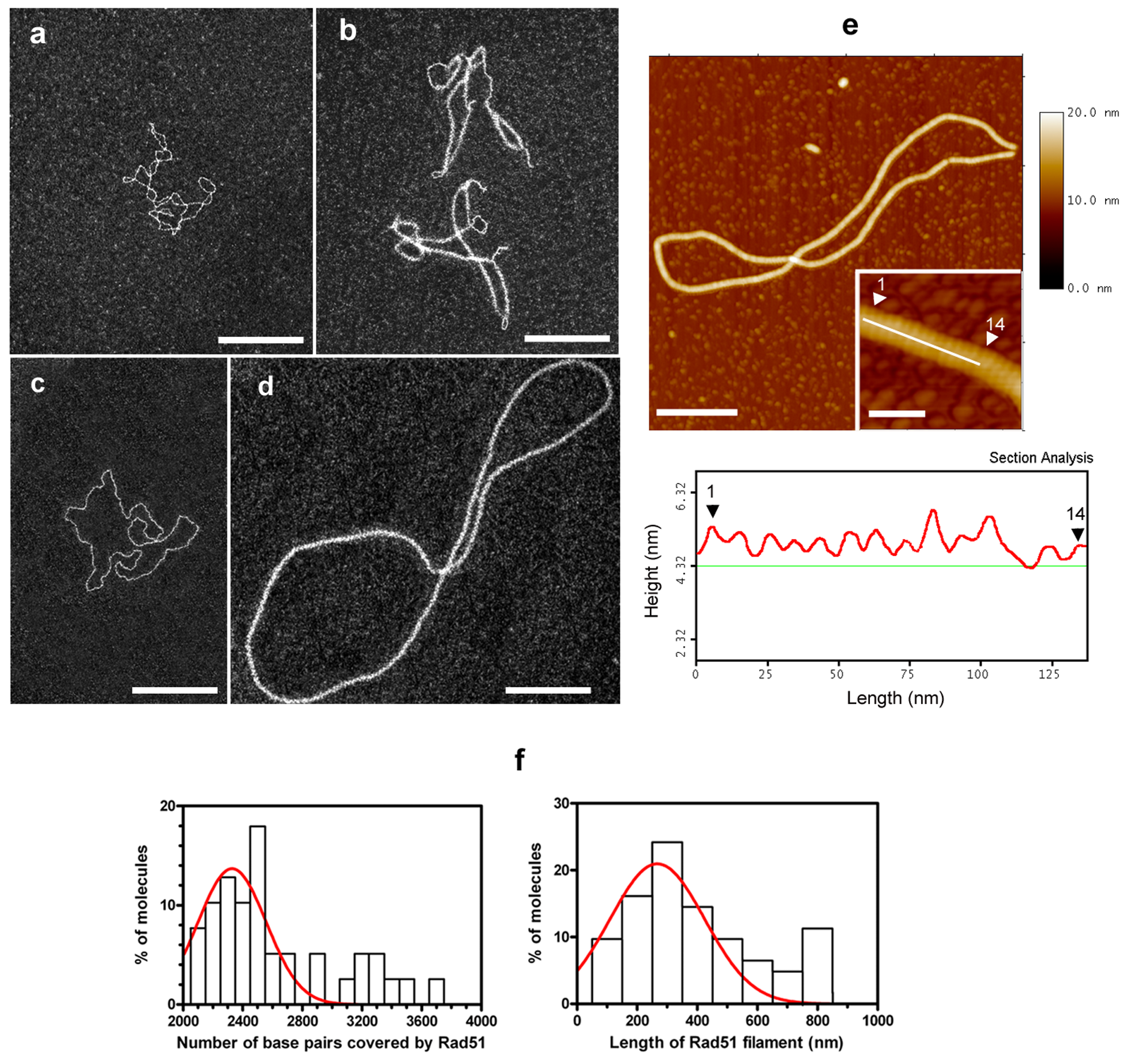

Supplement: Figure S2 — Rad51 polymerization on a naked plasmid. When Rad51 polymerizes on a closed circular naked plasmid (see naked control in a), high positive constraint concentrates in very densely positively supercoiled plectonemic regions (b). When relaxation is allowed during the reaction (c: see relaxed naked plasmid for comparison), filaments cover the whole plasmid (d). As in Fig. 4, AFM allows us to directly measure filament parameters (e: see insert in the picture and profile below). Total covering of the circular substrates and individual Rad51 filament lengths were obtained from image analysis of more than 200 molecules series from TEM and AFM pictures (f). The scale bars represent 200 nm (a–d), 250 nm (e) and 50 nm (inset). (10.07 MB TIF) [file pone.0003643.s002.tif]

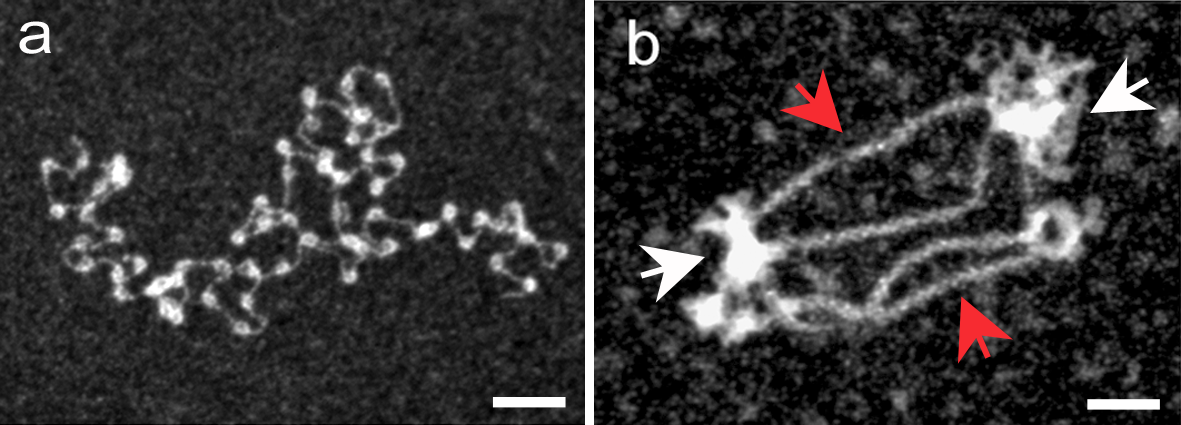

Supplement: Figure S3 — Rad51 polymerization on long native linear nucleosomal templates. When added on long native chromatin fibers (∼60 nucleosomes distributed on 11000 bp) (a), Rad51 polymerization induced chromatin remodeling of the same extent than the remodeling events observed on circular templates (b), confirming the robustness of this mechanism. Red and white arrows show Rad51 filament and nucleosome cluster, respectively. The scale bars represent 100 nm for all pictures. (1.52 MB TIF) [file pone.0003643.s003.tif]

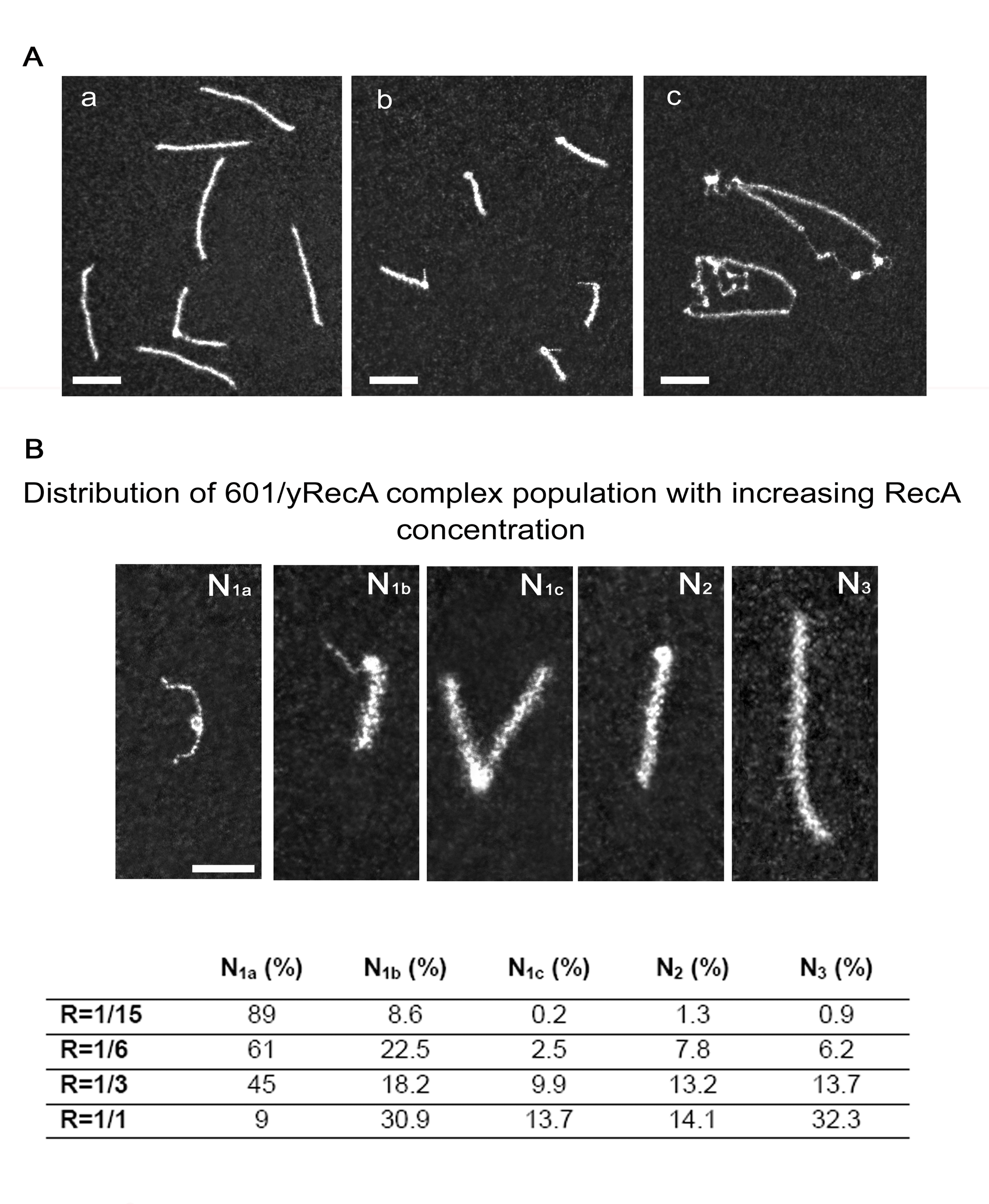

Supplement: Figure S4 — RecA polymerization also displays remodeling activity. A. Nucleosomal templates were reconstituted on a 601 positioning sequence located at the center of a 347 bp DNA fragment (a). Despite the high affinity of the 601 sequence, Rad51 polymerization shifts or ejects nucleosomes. The scale bars represent 50 nm for all pictures. B. Distribution of the 601/Rad51 complex population as a function of increasing [RecA]/[bp] ratio. All pictures were acquired at the same magnification and the scale bare represents 50 nm. (13.05 MB TIF) [file pone.0003643.s004.tif]
