# Supplementary material for: Rad51 Polymerization Reveals a New Chromatin Remodeling Mechanism
Source: PLoS One. 2008 Nov 4;3(11):e3643. doi: 10.1371/journal.pone.0003643 (PMC2574414; doi:10.1371/journal.pone.0003643)
Supplement: Table S1 — Sequence of primers used for PCR. (0.03 MB DOC) [file pone.0003643.s005.doc]

| Position on plasmid | Sequence (5'→3') |
| --- | --- |
| primers used for 5S fragment | |
| 210 | ACAGCTATGACCATGATT |
| 275 | CTGCAGGTCGACTCTAGA |
| primers used for construction of partially duplex DNA | |
| 2576 | CGACGCTCAAGTCAGAGG |
| b4016^c^ | GGATCTCAACAGCGGTAA |
| 3184 | TTAACGTGAGTTTTCGTTCC |
| b3416^c^ | CAGTGCTGCAATGATACCGC |
| 4016 | GGATCTCAACAGCGGTAA |

^c^ biotinylated nucleotides are shown in underlined

**Table S1: Sequence of primers used for PCR**
